# Both Sensorimotor Rhythm Neurofeedback and Self-Controlled Practice Enhance Motor Learning and Performance in Novice Golfers

**DOI:** 10.3390/bs13010065

**Published:** 2023-01-11

**Authors:** Zahra Pourbehbahani, Esmaeel Saemi, Ming-Yang Cheng, Mohammad Reza Dehghan

**Affiliations:** 1 Department of Motor Behavior and Sport Psychology, Faculty of Sport Sciences, Shahid Chamran University of Ahvaz, Ahvaz 6135783151, Iran; 2 School of Psychology, Beijing Sport University, No. 48 Xinxi Road, Haidian District, Beijing 100084, China; 3 Department of Sport Physiology, Faculty of Sport Sciences, Shahid Chamran University of Ahvaz, Ahvaz 6135783151, Iran

**Keywords:** SMR neurofeedback, yoked practice, golf putting task, additive effect, motor learning

## Abstract

A major concern voiced by motor behavior scientists is to find useful practice techniques that can be effective in improving motor learning and performance. Neurofeedback and self-controlled practice are among the techniques that have recently drawn attention from specialists in this area. The present study examined the additive and individual effects of sensorimotor rhythm (SMR) neurofeedback as well as self-controlled practice on motor learning and performance in novice golfers. In this semi-empirical study, forty adults (20 females, Mean_age_ = 26.10, SD = 5.56 years) were conveniently selected and randomly assigned to four groups: (1) neurofeedback/self-controlled practice, (2) neurofeedback/yoked practice, (3) sham/self-controlled practice, and (4) sham/yoked practice. The participants performed golf putting task in four stages, namely pretest (12 trials), intervention (one day after pretest; 6 sessions, 36 trails each), post-test (one day after intervention; 12 trials), and follow-up (two weeks after interventions; 12 trials). In addition, the participants had their EEG (SMR wave in Cz point) recorded during pretest, post-test, and follow-up. The results indicated that, although no additive effect was observed for the two practices during different stages of the experiment (*p* > 0.05), in acquisition and post-test stages, SMR neurofeedback and self-controlled practice independently facilitated golf putting (*p* ≤ 0.05). However, in the follow-up test, only the neurofeedback practice maintained its positive effects (*p* ≤ 0.05). The results also showed that participation in SMR neurofeedback practice can enhance the power of the SMR wave (*p* ≤ 0.05), regardless of the type of the self-controlled practice used. In sum, the two practice techniques seem to be independently effective in facilitating motor learning in instructional settings, particularly for golfers.

## 1. Introduction

A major concern, constantly cited by motor behavior scientists and coaches, is to find useful practice techniques that can be effective in improving motor learning and performance. One such technique that has recently drawn considerable attention from scientists and researchers is neurofeedback practice [1,2,3]. In this method, by training his/her mind, an individual learns to regulate brain activities towards a better, optimal performance [4,5]. In other words, the neurofeedback technique develops the nervous system for the right pattern or activity by increasing or decreasing different brainwave activities in certain regions of the cerebral cortex over several sessions. Essentially, the neurofeedback technique can suppress the waves beyond a standard range or enhance the waves below that range [3]. Researchers believe that neurofeedback can improve or modify disturbed brainwave patterns through operant conditioning while providing feedback on brain activities [6].

The psychomotor efficiency hypothesis [7] states that suppression of task-irrelevant cognitive and motor processes (e.g., reduced neural motor noise) can refine motor skills [3]. The hypothesis was supported by studies that used novice–expert models where experts in different tasks including golf putting or shooting reported global reduction in their cerebral cortex activity compared to novice athletes [8,9].

Furthermore, previous studies have established effects of neurofeedback practice on improvement of performance and learning in variety of sports. For example, Landers et al. [10] demonstrated that neurofeedback practices, when carried out correctly, resulted in improved motor performance in skilled archers (e.g., increased slow potentials of the cerebral cortex in the left temporal lobe), while feedback practices wrongly carried out (i.e., accompanied with suppression and reinforcement in the wrong area which involved increased slow potentials of the cerebral cortex in the right temporal lobe) disrupted performance in skilled archers. In other words, the application of different correct forms of neurofeedback practice significantly enhanced performance levels and psychological indices in athletes [11].

Many studies that used electroencephalography (EEG) to examine dynamics of neural activities in the cerebral cortex and recorded various brainwaves including theta (4–8 Hz), alpha (10–12 Hz), beta (22–15), and sensorimotor rhythm (12–15) at different points reported that all these waves can be useful in achieving a better understanding of improvements in cognitive–motor processes involved in motor preparation [12,13]. For example, Hatfield, Landers, and Ray [14] reported that, as elite shooters approach the moment of pulling the trigger, a gradual increase occurs in the left temporal alpha wave. Further studies examined enhancement and/or suppression of brainwaves such as alpha at the left temporal region [15,16,17], theta [5], and sensorimotor rhythm [18]. Most of these studies have shown that enhancement or suppression of these brainwaves at different regions of the cerebral cortex may have different impacts on cognitive and motor performance improvement for different individuals (for a review, see [19]).

Among the various neurofeedback protocols used to inhibit or enhance brainwaves, the sensorimotor training protocol was proposed as an effective method in enhancing motor performance [3,20]. Cheng et al. [18] found a higher sensorimotor power in Cz point associated with skilled performance of goal-oriented tasks. In addition, their findings confirmed the causal relation between sensorimotor rhythm (SMR) and performance in such tasks as golf putting, indicating that high levels of sensorimotor rhythm in Cz point improved performance through neurofeedback, resulting in better performance of golf putting by skilled golfers [20]. In sum, enhanced advantages of sensorimotor rhythm neurofeedback practice can be attributed to improved regulation of sensory and sensorimotor pathways which, in turn, may lead to a more effective allocation of attention [21] and better processing of task-relevant stimuli. However, while this relation has been established for skilled performance, questions still remain regarding the connection between enhanced sensorimotor waves and motor learning in novice golfers. Thus, one goal of the present study is to examine effects of neurofeedback practice in connection with enhancing sensorimotor waves in the Cz point and how it may influence motor performance and learning of novice golfers.

In addition, the learning environment is another factor that influences improvements in motor performance and learning. A notable characteristic of a learning environment is the autonomy and freedom of choice given to a learner in that environment [22,23,24]. For example, motor skill learning can be improved by asking learners to have a choice during training; a condition often referred to as self-controlled practice [22]. The choice provided to the individual (self-control) or the right to choose is an essential psychological [25,26] and biological [27] need which is inherently rewarding. For example, Iwatsuki, and Otten [28] indicated that providing choice to participants enhanced motor learning in learners.

Preliminary evidence linked improved learning resulting from enhanced cognitive performance to self-controlled environments where participants performed choices related to training and practice [29]. However, do these positive effects remain in force when participants carry out choices that are irrelevant to training and practice content? In this regard, Lewthwaite et al. [30] showed that giving learners a choice (even irrelevant choices) about the color of the golf ball in training sessions enhanced their learning in golf putting task for the participants in the choice group compared to a yoked group where participants were not given a choice. Therefore, another role that can be played by self-controlled environments in enhancing motor learning concerns motivational effects of such environments. According to the optimal theory of motor learning, providing learners with a choice supports their essential psychological need for autonomy, thereby directly enhancing their motor performance and learning by improving their intrinsic motivation [31]. Thus, another goal pursued by the present study is to examine how self-control practice can influence motor performance and learning in golf putting for novice golfers.

One technique used for improving quality of training programs is to integrate the effects of two or more independent training techniques into a single practice session [31]. Studies have shown that these additive effects can, in some cases, be more effective than individual training techniques [32]. To the best of the authors’ knowledge, no study so far has examined combined effects of neurofeedback practice combined with self-control practices on motor learning, particularly when a sensorimotor brainwave protocol is used for neurofeedback practice. Previous studies have only investigated individual effects of either neurofeedback practice [3,20,33,34] or self-controlled practice [28,29,30] on motor learning and performance. Therefore, another goal of the present study is to investigate the additive effects of these two practice techniques and to compare these additive effects to the respective individual effects of each method on motor performance and learning in novice golfers. The authors have hypothesized that this additive effect may go beyond the individual effects of each technique, positively influencing motor performance and learning in golf putting.

## 2. Materials and Methods

### 2.1. Participants

The minimum detectable effect was determined through statistical power analysis for mixed analysis of variance by repeated measurements for calculating sample size using G*Power 3.1 [35]. Based on previous studies in this area [36], the analysis was carried out using the following values: α = 0.05, statistical power = 0.80, effect size = 0.70 (equivalent to η_p_^2^ = 0.33), number of groups = 4, and number of repeated measures = 6. The minimum sample size was found to be N = 28. To account for data attrition, eventually 40 individuals were sampled into the study. The participants were undergraduate students (20 females, mean age = 26.10, SD = 5.56) selected through convenient sampling and randomly assigned into four groups: (1) neurofeedback/self-controlled practice, (2) neurofeedback/yoked practice, (3) sham/self-controlled practice, and (4) sham/yoked practice. Based on our inclusion criteria for this study we included individuals who were (1) right-handed, (2) novice in the golf putting task, and (3) physically and mentally healthy. We excluded individuals who (1) were unwilling to continue the activity or (2) sustained any injury during practice sessions.

### 2.2. Study Design

**Figure 1:**
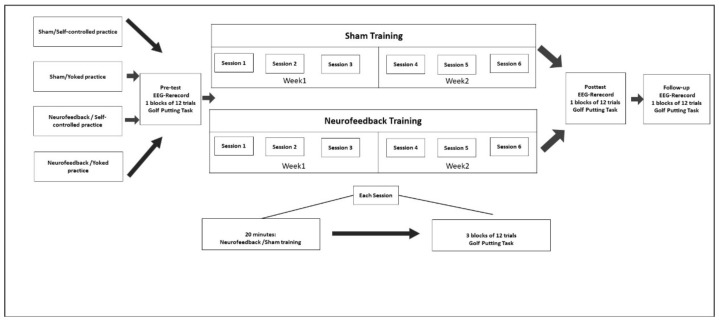
Experimental flowchart.

The present study was a semi-empirical study with four phases of pretest, intervention, post-test, and follow-up (Figure 1). The study design was developed based on the Declaration of Helsinki and approved by Shahid Chamran University’s Ethics Committee (Approval Code: EE/1400.2.24.32887/Scu.ac.ir; Approval Date: 14 May 2021). All participants completed an informed consent form prior to taking the tests.

### 2.3. Measurement Apparatus

#### 2.3.1. The Golf Putting Task

**Figure 2:**
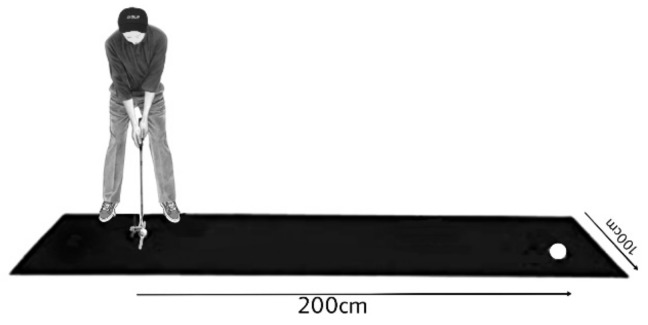
The schematic illustration of the golf putting task.

The task given in this study involved a turf field, a putter, and a standard golf ball (similar to previous studies, [37]). The participants used a right-hand standard putter to hit a standard golf ball (diameter = 4.27 cm) on a 400 cm × 100 cm turf field which contained a standard hole (diameter = 10.8 cm) as the target which was placed 200 cm from the starting point that was indicated in front of the participants using a white strip having a width of 5 cm. The goal was to stop the ball as close as possible to the target center. The distance from the target center to the ball edge after each trial was recorded as an indicator of radial error (Figure 2).

#### 2.3.2. Neurofeedback Apparatus

**Figure 3:**
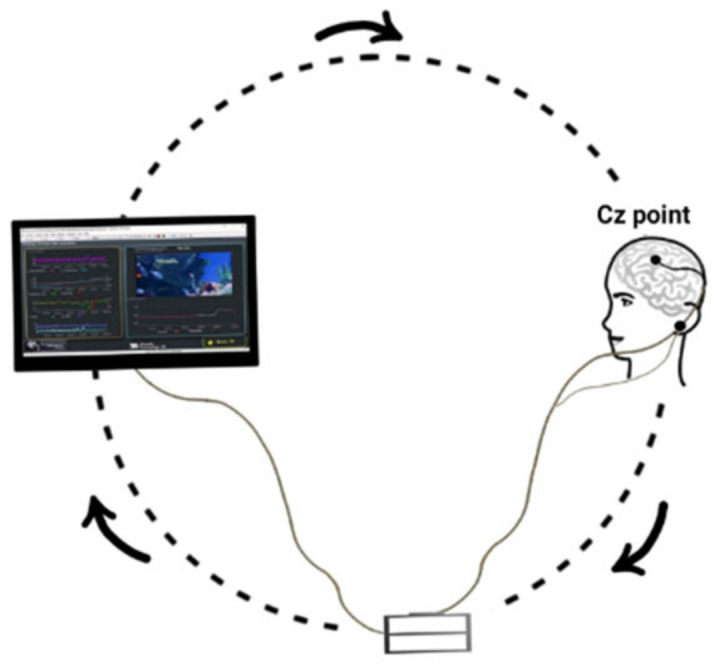
The schematic illustration of the neurofeedback apparatus.

ProComp5 Infiniti-5 Channel (Thought Technology, Montreal, QC, Canada) was employed to provide neurofeedback practice. The neurofeedback practices were completed using BioGraph Infiniti. First, EEG cap was used to measure SMR waves obtained from the participants by getting a base record from cerebral cortex activity at Cz. To measure and record SMR waves, an electrode was placed at Cz according to the 10–20 international system. Two electrodes as the ground electrode (left ear) and the reference electrode (right ear) were placed on either earlobe (Figure 3). In administering the neurofeedback practices, once the electrodes were placed on the head, the participant faced a computer monitor to watch an animation which was prepared based on the common protocols to enhance the SMR wave [20].

### 2.4. Procedure

In this semi-experimental study, forty adults were conveniently selected and randomly assigned to four groups. The study involved four stages: pretest, intervention (one day after pretest), post-test (one day after intervention), and follow-up (two weeks after intervention). In three stages, namely pretest, post-test, and follow-up, an initial base EEG record was obtained from the participants with open and closed eyes for two minutes to assess the SMR wave. In the pretest, all participants took a 12-trial instruction-free test using a white standard golf ball and had their radial errors recorded in each trial. The intervention stage involved 6 sessions (each consisting of 20 min of neurofeedback/sham practices followed by golf putting for 3 blocks of 12 trials). The rest time between trials was around 5 min. Prior to neurofeedback practice or sham conditions, the participants were given the following instructions: “The laptop in front of you will show you an animation which is related to your brain activity. When you reach a certain level of brain activity, the animation size will increase and it will start beeping at the same time; otherwise, the animation size will be small. This shows that you are now focused on the task. You are asked to follow the instructions and do this for 20 min”. Before intervention, all participants were asked to confirm that the instructions were not confusing. In the intervention stage, the participants in the neurofeedback + self-controlled practices first practiced the neurofeedback practices with the SMR enhancement protocol at Cz for 20 min. Then, following a 2-min break, they performed the golf putting task in three blocks of 12 trials: before each trial, they were given the choice to pick one of the three standard golf balls colored in yellow, red, or blue to complete the putting task. In the neurofeedback + yoked practice group, the participants received the same neurofeedback practices as the first group, only in the yoked practices each participant was yoked to a participant from the first group and putted the ball using the same color as the one used previously in the self-controlled group. In the third group, i.e., sham + self-controlled practice, the participants were first trained by watching a recorded video for 20 min while thinking that it was them who controlled the animation. Then, they performed 3 blocks of 12 putting trails using self-controlled practice (similar to the first group). The participants in the last group, i.e., sham + yoked practice, first received the same conditions as the third group for 20 min, and then performed 3 blocks of 12 putting trials (similar to the second group, i.e., the yoked group).

### 2.5. Data Analysis

Statistical mean and standard deviation values were used to analyze the data. Furthermore, inferential analysis was conducted using 2 (neurofeedback/sham) × 2 (self-control/yoked) × 6 (training sessions) mixed ANOVA by repeated measure over the last factor for putting accuracy as the dependent variable in the intervention stage. For post-test and follow-up, data were analyzed through 2 (neurofeedback/sham) × (self-control/yoked) two-way ANOVA. Moreover, the data concerning SMR wave power was analyzed using 2 (neurofeedback/sham) × 2 (self-control/yoked) × 3 (pretest, post-test, follow-up) with repeated measures on the last factor. In addition, pairwise comparisons of variables were conducted using the Bonferroni post-hoc test. The significance level for the test was set at 0.05 and the data were analyzed in SPSS v. 24.

## 3. Results

The initial findings confirmed all assumptions needed for inferential statistics, i.e., the assumption of equal variances as well as data normality. Table 1 presents participants’ characteristics together with dependent variables in the pretest for the participants in all four experimental groups. As seen in this table, all groups received similar scores on the dependent variables in the pretest.

### 3.1. Golf Putting

#### 3.1.1. Acquisition

**Figure 4:**
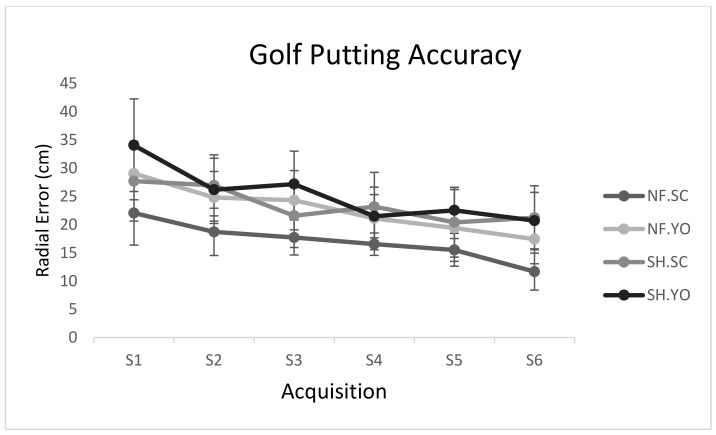
Line chart of golf putting accuracy during acquisition stage. Error bars represent the standard deviation. NF. SC: neurofeedback/self-controlled practice; NF. YO: neurofeedback/yoked practice; SH. SC: sham/self-controlled practice; SH. YO: sham/yoked practice.

The initial findings confirmed the assumption of Mauchly’s sphericity test (*p* > 0.05). Thus, the original values were reported. The results of 2(neurofeedback/sham) × 2(self-control/yoked) × 6(training sessions) mixed ANOVA with repeated measures on the factor indicated significant main effects of sessions (F(5, 180) = 40.69, *p* = 0.0001, η_p_^2^ = 0.53), session-group interaction (self-control/yoked; F(5, 180) = 3.27, *p* = 0.007, η_p_^2^ = 0.08), the main group effect (neurofeedback/sham; F(1, 36) = 13.32, *p* = 0.001, η_p_^2^ = 0.27), and the main group effect (self-control/yoked; F(1, 36) = 8.96, *p* = 0.005, η_p_^2^ = 0.19), while other effects were not found to be significant. Post-hoc test results demonstrated that all groups experienced a significant improvement in their performance during the intervention, with the neurofeedback group (19.2 ± 58.78) outperforming the sham group (24.2 ± 43.77), regardless of the presence of self-control or yoked conditions. In contrast, the self-control group (20.2 ± 26.77) outperformed the yoked group (24.2 ± 01.78), regardless of the presence of neurofeedback or sham practice (Figure 4).

#### 3.1.2. Post-test

**Figure 5:**
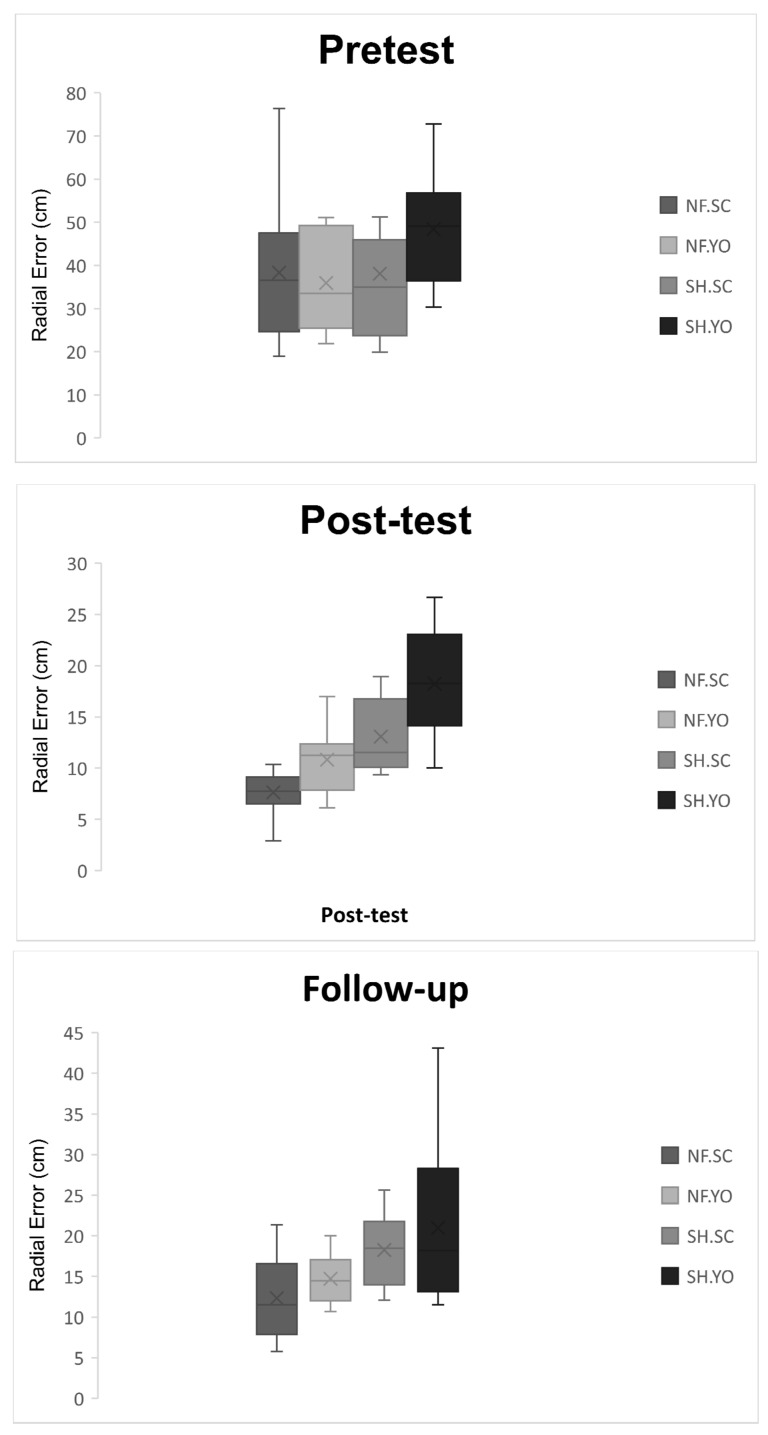
Boxplot of golf putting accuracy during pretest, post-test, and follow-up. Crosses in each box represent the mean. NF.SC: neurofeedback/self-controlled practice; NF. YO: neurofeedback/yoked practice; SH.SC: sham/self-controlled practice; SH. YO: sham/yoked practice.

The results from 2(neurofeedback/sham) × 2(self-control/yoked) two-way ANOVA on the post-test scores showed that the main group effect (neurofeedback/sham; F(1, 36) = 30.48, *p* = 0.0001, η_p_^2^ = 0.45) as well as the main group effect (self-control/yoked; F(1, 36) = 12.88, *p* = 0.001, η_p_^2^ = 0.26) were significant, while other effects were not found to be significant. Post-host test results showed that the neurofeedback group (9.2 ± 22.60) outperformed the sham group (15.2 ± 65.60), regardless of the presence of self-control or yoked condition. In contrast, the self-control group (10.2 ± 35.60) outperformed the yoked group (14.2 ± 53.60), regardless of the presence of neurofeedback or sham practice (Figure 5).

#### 3.1.3. Follow-up

The results from 2(neurofeedback/sham) × 2(self-control/yoked) two-way ANOVA on the follow-up scores showed that only the main group effect (neurofeedback/sham; F(1, 36) = 9.44, *p* = 0.004, η_p_^2^ = 0.20) was significant and no other significant effect was reported. The post-hoc test results indicated that the neurofeedback group (13.4 ± 53.42) outperformed the sham group (19.4 ± 62.42), regardless of the presence of self-control or yoked conditions (Figure 5).

### 3.2. SMR Wave Power

#### 3.2.1. Open Eye Condition

**Figure 6:**
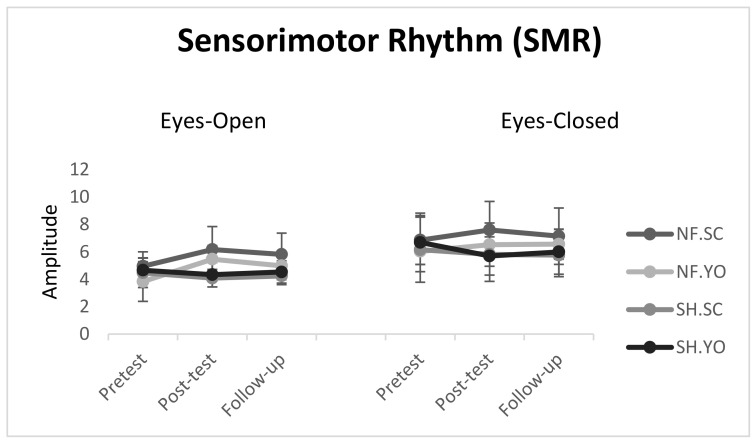
Line chart of Sensorimotor Rhythm (SMR) power during pretest, post-test, and follow-up. Error bars represent the standard deviation. NF.SC: neurofeedback/self-controlled practice; NF. YO: neurofeedback/yoked practice; SH.SC: sham/self-controlled practice; SH. YO: sham/yoked practice.

The initial findings did not support the assumption of Mauchly’s sphericity test (*p* > 0.05). Therefore, Greenhouse–Geisser values were reported instead. The results of 2(neurofeedback/sham) × 2(self-control/yoked) × 3(pretest, post-test, follow-up) mixed ANOVA with repeated measure on the last factor indicated significant main effects for the experimental stages (pretest, post-test, follow-up; (F(1.44, 51.99) = 9.96, *p* = 0.001, η_p_^2^ = 0.21)) as well as the group effect (neurofeedback/sham; F(3, 36) = 6.45, *p* = 0.015, η_p_^2^ = 0.15)). The interaction effect for the three stages (pretest, post-test, follow-up) and the group (neurofeedback/sham) was also reported to be significant (F(1.44, 51.99) = 26.45, *p* = 0.0001, η_p_^2^ = 0.42)). However, other effects were not found to be significant. The results from the post-hoc tests showed that in both post-test (6.1 ± 17.68) and follow-up (5.1 ± 81.56), the neurofeedback group outperformed the sham group (5.1 ± 46.00; 4.98 ± 1.01) in terms of SMR wave power, regardless of the presence of self-control or yoked practice (Figure 6).

#### 3.2.2. Closed Eye Condition

The initial findings did not support the assumption of Mauchly’s sphericity test (*p* > 0.05). Therefore, Greenhouse–Geisser values were reported instead. The results of 2(neurofeedback/sham) × 2(self-control/yoked) × 3(pretest, post-test, follow-up) mixed ANOVA with repeated measure on the last factor indicated significant effects for interaction of the stages (pretest, post-test, follow-up) and group (neurofeedback/sham) (F(2, 72) = 14.08, *p* = 0.0001, η_p_^2^ = 0.28). However, other effects were not found to be significant. The results from the post-hoc tests showed that only in the post-test (7.2 ± 60.08), the neurofeedback group outperformed the sham group (6.1 ± 53.58) in terms of SMR wave power, regardless of the presence of self-control or yoked practice conditions (Figure 6).

## 4. Discussion

The present study was conducted to examine the additive and individual effects of the SMR-based neurofeedback as well as self-controlled practice on motor learning and performance in novice golfers. The findings indicated that, while during the different experimental stages no additive effect was observed for the two practice techniques, in the acquisition stage, the SMR-based neurofeedback practice and the self-control practice independently facilitated acquisition of golf putting in novice learners. In addition, during the post-test conducted one day after the intervention, the two techniques were able to positively influence golf putting. However, in the follow-up conducted two weeks after the completion of the intervention sessions, only the neurofeedback technique maintained its positive effect while the self-control technique failed to create persisting effects. Furthermore, the results demonstrated that participation in the SMR-based neurofeedback practices can enhance SMR wave power, regardless of the type of self-control practice. 

The findings concerning effectiveness of SMR neurofeedback practices in motor performance and learning are consistent with a number of previous studies in this area [4,8,19,20]. Although these practices have been widely conceptualized for treating a number of cognitive–motor disorders and as techniques to reduce impulsivity/hyperactivity, [38] or in a more general sense to enhance attention processing [39], they have also been reported to be linked to the optimization of skilled motor performance of such skills as golf putting, dart throwing, and shooting [8,19,20,40]. The study findings also demonstrated facilitative effects of SMR neurofeedback practices in athletes and healthy individuals. For example, Cheng et al. [20] showed that a course of SMR neurofeedback practices can improve performance in skilled golfers. In addition, Norouzi et al. [41] reported this improvement in bimanual coordination for children with ADHD. One possible reason for the positive influence of this training protocol can be attributed to enhanced perceptual sensitivity as well as the elimination of, or decrease in, motor variability following application of SMR neurofeedback practices [42]. Furthermore, in line with the psychomotor efficiency hypothesis [7], in the present study, it seems that the participants in the neurofeedback group were able to facilitate their motor learning by suppressing task-irrelevant cognitive and motor processes; for example, through reduced neural motor noise [3]. It can be concluded that perhaps, in the present study, the participants who practiced in the neurofeedback group experienced reduced error and motor variability due to enhanced neural communications and suppressed task-irrelevant motor–cognitive processes. This, in turn, improved their motor performance and learning. A number of studies even argued that the positive effects of SMR neurofeedback practices on motor performance and learning are stronger than other commonly used neurofeedback protocols, including training of the alpha rhythm [19,20].

Studies have shown a significant positive connection between higher SMR wave power and motor performance [8]. For example, Cheng et al. [8] found that skilled dart players who outperformed novice players actually exhibited higher levels of SMR wave power in their cerebral cortex. In addition, neurofeedback practice can influence neuroplasticity and alter neural activity of the brain. Consistent with Gong et al. [19], the findings of the present study showed that SMR wave power at rest changed under open and closed eye conditions following zero-self-control neurofeedback practices. Although this finding cannot directly demonstrate increased neuroplasticity in the nervous system, it can point to improved behavioral measures [19]. Therefore, the enhanced motor learning and performance in the participants of this study can be attributed to the fact that SMR neurofeedback practices improved motor learning and performance by increasing SMR wave power in individuals and by enhancing adaptive regulation of motor–cognitive processing during preparation [8].

Furthermore, previous studies often pointed to a considerable increase in attention with increased SMR [20,42]. Mann et al. [43] suggested that SMR is negatively correlated with relay activity in the sensorimotor cortex, indicating an inhibition of transmission of somatosensory information during SMR activity. In sum, the present findings can be interpreted as supporting evidence for the idea that effects of SMR neurofeedback practice on sensorimotor control have advantages that go beyond direct influence on impulsive aspects of attention [42]. In other words, sensorimotor pathways reduce processing interference, leading to more efficient, higher-level attention processing. This means a cognitive integration of task-relevant stimuli. In the present study, the participants were novice learners who were learning the golf putting task and simultaneously receiving the SMR neurofeedback protocol. Research has shown that athletes respond differently to neurofeedback training protocols than non-athletes [44]. Therefore, it seems that one of the reasons for the effectiveness of neurofeedback practices in the present study is related to this issue. On the other hand, in the present study, the participants performed neurofeedback practices during a two-week period (three sessions per week). Research has shown that doing three sessions a week can more effectively show the positive effects of neurofeedback than doing two sessions of neurofeedback training a week [45]; therefore, another possible reason for the positive effect of the neurofeedback protocol in the present study could be related to doing three sessions (and not less) per week.

On the other hand, the second part of our findings confirms that providing the participants with a choice, however small (such as choosing the ball colo), during golf putting can positively influence motor learning. The participants who were given a choice on ball color outperformed the participants in the yoked group in terms of motor learning and performance, regardless of the presence or absence of neurofeedback practices. This finding is consistent with many studies in this area [22,23,24,28,29,30,46]. For example, An et al. [46] showed that self-control practice can be an efficient technique to facilitate motor performance and learning in golf putting task. They concluded that providing the learner with a small choice during golf putting practice results in motivational advantages that enhance learning and self-confidence and leads to more positive emotional responses. It can be argued that the participants in the present study were able to improve their motor learning due to their increased level of motivation in the self-control group. However, caution must be taken in presenting such an argument since the present study did not directly measure the participants’ motivations.

It seems that providing learners with choice and autonomy enables them to test different motor and cognitive strategies [29] and allows them to control these strategies based their needs. This is often useful in approving the learner’s successful performance [30]. In fact, self-control practice provides a learner with an opportunity for a more creative practice by which to discover motor strategies [47], less stress and improved well-being [48], and more energy and liveliness [49] since it can meet an essential psychological need [25,26]. Therefore, another reason for better learning in the self-control practice group can be attributed to a more efficient processing of cognitive and motor information [29].

In addition, according to the optimal theory of motor learning in a motor learning process, one attentional variable (using external attention) and two motivational variables (providing a choice and enhanced hope levels for learners) can facilitate motor learning and performance [31]. These variables, whether individually or collectively, can facilitate release of dopamine in the nervous system [50,51], which has been shown directly and indirectly to enhance movement effectiveness and efficiency [52]. Increased release of dopamine caused by positive experiences promotes neural communications [53] which can act as a mechanism to improve motor learning [54]. Thus, this mechanism can also be used to explain why the participants in the self-control practice group demonstrated better motor performance and learning in the present study.

Like many other studies, the present study has a number of limitations. One limitation stems from the fact that the present study lacked an actual control group, i.e., one excluded from the intervention. Another limitation is that the present study did not measure such variables as intrinsic motivation or positive emotions. Assessment of these variables can be helpful in identifying the mechanisms involved in self-control practice; it is therefore recommended that future studies take this into account.

## 5. Conclusions

As a conclusion, the findings of this study demonstrated individual independent effects of neurofeedback practice and self-control practice on motor performance and learning in golf putting. However, our study failed to report any combined effect. Thus, it seems that both methods can individually and independently facilitate motor learning in instructional environments, particularly for golfers.

## Figures and Tables

**Table 1:** Participant characteristics and research variables.

Personal Characteristics and Research Variables	Groups (M ± SD)				Significance Level
	Neurofeedback/Self-Control	Neurofeedback/Yoked	Sham/Self-Control	Sham/Yoked	
N	10	10	10	10	-
Age (year)	23.3 ± 80.42	25.3 ± 00.65	27.6 ± 80.05	27.6 ± 80.69	0.22
Height (cm)	169.9 ± 60.24	171.9 ± 00.93	172.12 ± 90.17	169.9 ± 70.16	0.87
Weight (pretest)	64.16 ± 10.12	63.20 ± 90.82	72.11 ± 90.37	65.11 ± 90.74	0.53
BMI (kg/m^2^; pretest)	21.3 ± 95.29	21.5 ± 53.27	24.3 ± 47.80	22.2 ± 80.96	0.36
Radial error (cm; pretest)	38.17 ± 32.42	35.11 ± 91.14	38.17 ± 08.94	48.13 ± 41.21	0.27
SMR power (open eyes; pretest)	4.1 ± 95.05	3.1 ± 83.45	4.1 ± 47.07	4.0 ± 66.90	0.17
SMR power (closed eyes; pretest)	6.1 ± 86.79	6.0 ± 4.96	6.2 ± 16.38	6.2 ± 96.14	0.72

## Data Availability

Not applicable.
